# Effects of eHealth Literacy on General Practitioner Consultations: A Mediation Analysis

**DOI:** 10.2196/jmir.6317

**Published:** 2017-05-16

**Authors:** Peter Johannes Schulz, Mary Anne Fitzpatrick, Alexandra Hess, Lynn Sudbury-Riley, Uwe Hartung

**Affiliations:** ^1^ Institute of Communication and Health Faculty of Communication Science Università della Svizzera italiana Lugano Switzerland; ^2^ Waikato Management School University of Waikato Hamilton New Zealand; ^3^ School of Communication, Journalism and Marketing Massey University Auckland New Zealand; ^4^ University of Liverpool Management School Liverpool United Kingdom

**Keywords:** health literacy, eHealth literacy, health empowerment, information seeking, health care utilization, baby boomers

## Abstract

**Background:**

Most evidence (not all) points in the direction that individuals with a higher level of health literacy will less frequently utilize the health care system than individuals with lower levels of health literacy. The underlying reasons of this effect are largely unclear, though people’s ability to seek health information independently at the time of wide availability of such information on the Internet has been cited in this context.

**Objective:**

We propose and test two potential mediators of the negative effect of eHealth literacy on health care utilization: (1) health information seeking and (2) gain in empowerment by information seeking.

**Methods:**

Data were collected in New Zealand, the United Kingdom, and the United States using a Web-based survey administered by a company specialized on providing online panels. Combined, the three samples resulted in a total of 996 baby boomers born between 1946 and 1965 who had used the Internet to search for and share health information in the previous 6 months. Measured variables include eHealth literacy, Internet health information seeking, the self-perceived gain in empowerment by that information, and the number of consultations with one’s general practitioner (GP). Path analysis was employed for data analysis.

**Results:**

We found a bundle of indirect effect paths showing a positive relationship between health literacy and health care utilization: via health information seeking (Path 1), via gain in empowerment (Path 2), and via both (Path 3). In addition to the emergence of these indirect effects, the direct effect of health literacy on health care utilization disappeared.

**Conclusions:**

The indirect paths from health literacy via information seeking and empowerment to GP consultations can be interpreted as a dynamic process and an expression of the ability to find, process, and understand relevant information when that is necessary.

## Introduction

### Health Literacy and the Utilization of the Health Care System

The high attention health communication research pays to health literacy is grounded in a core group of factors positively related to health literacy. Among these factors are self-reported health status [1-3], participation in prevention and screening [4,5], frequency of exercise [6,7], a healthy diet [8,9], better management of chronic diseases [10,11], and even lower mortality for individuals with serious conditions [6,12].

Another key factor on this list is the utilization of the health care system; that is, visits to one’s physician or general practitioner (GP) and other health care professionals, visits to accident and emergency facilities, admissions to hospitals, and various health care treatments. The usual implication is that high levels of health literacy are associated with low levels of using the services of the health care system, assuming that lower levels of utilization are a good thing. This assumption is based on the fact that higher utilization of the health care system is generally associated with higher costs [9,13-16].

Studies provide strong empirical evidence for the inverse relationship of health literacy and health care system utilization in different countries [17-21], across different age-groups [21,22], as well as across different patient groups [23-26]. Most researchers seem to agree that health literacy affects health care system utilization; however, there are several explanations proposed for this effect. For instance, people with low health literacy skills are understood to have limited abilities to access and understand health information, and to make decisions about their health. According to Baker et al [22], these people delay seeking care for serious health problems and have poor self-management skills; therefore, they eventually have higher rates of hospitalization and more visits to emergency clinics, both for treatment of serious conditions and for chronic care or conditions that could be more effectively managed by visiting a regular GP and early intervention [18]. Those with inadequate health literacy might not be aware of health services such as screening tests and their role in disease prevention and early diagnosis, and consequently use less preventive care [20,27] and rely more on prescription therapy than prevention [28]. Other researchers contend that patients with low health literacy can feel ashamed or distrustful of the health care system [29,30] and use more emergency medical care because they do not have a regular GP or health professional [31].

Several researchers explain the inverse correlation by findings that individuals with high health literacy know more about diseases and self-care, engage in positive health behaviors, use more preventive health care, and have better compliance with medication regimens [20,32]. Rasu et al [28] agree that high literacy likely goes along with higher use of preventive care and thus, in the long run, with lower utilization; however, upward they point out that the correlation can also be explained by the increasing use of electronic media such as the Internet to help manage symptoms and conditions.

We note that as more studies investigate the relationship between health literacy and health care system utilization in different contexts, important inconsistencies are also being reported. Some studies find no statistically significant evidence of a relationship. For instance, health literacy was not independently associated with utilization of the health care system by adults in Iran [33]. Similarly, researchers did not find health literacy was a barrier to service utilization for adults with addiction [34] or for caregivers of children with asthma [35]. Finally, some results focus specifically on the association between health literacy and utilization of a specific service within the health care system. For example, health literacy in adult patients presenting to emergency care clinics (Atlanta, United States) was related to hospitalization but not to visits with one’s physician [1], whereas another study of respondents with heart failure showed no association between health literacy and hospitalization specifically [36]. Considered together, such findings highlight the urgent need for more research that investigates the nature of the health literacy-utilization correlation.

This article aimed at contributing to meet that need for a special subset of the two concepts: eHealth literacy rather than general health literacy and number of visits to one’s GP as one aspect of utilization of the health system. We chose to study eHealth literacy because the Internet has become one of the primary sources for health information [37], and versatility in using this source can be expected to affect health decisions, such as the decision between seeking consultation and trying to help yourself.

### The Relationship between Health Literacy and eHealth Literacy

We use the broad definition of health literacy cited frequently in the literature: “Health literacy is the degree to which individuals have the basic capacity to obtain, process, and understand basic information and services needed to make appropriate health decisions” [38]. Historically, the ability to read, comprehend, and act on health-related information related primarily to material provided by one’s GP or health care organizations. However, broader contemporary definitions of health literacy acknowledge patients initiating their own searches for information, then processing that information, and applying it in their interactions with health care practitioners and with the health care system [2,37,39,40]. A consumer behavior with important implications for patient empowerment, patient responsibility, and self-care, patient-initiated information search has been facilitated by the Internet, which continues to make increasing amounts of health information available through an expanding range of Web-based channels and communication technologies. Today, the Internet is a major source of health information [41] and the impact of Web-based information on patient health behaviors is increasing [42].

The corresponding abilities and skills needed by people to educate themselves on health matters using the Internet are brought together in the concept of eHealth literacy: the “foundational skill set that underpins the use of information and communication technologies (ICT) for health” [43]. Regarded as a “metaliteracy” [44], eHealth literacy combines both specific and general forms of literacy. Health literacy is one of the three specific sets of skills comprising eHealth literacy, along with computer literacy and science literacy. The general forms include traditional, information, and media literacies. Therefore, whereas health literacy certainly features prominently in eHealth literacy, nevertheless it is just one of six essential literacies. The eHealth literacy concept thus reflects the complexity inherent in the use of Internet information and technologies for health compared with the use of offline resources.

Finally, with health literacy and eHealth literacy presented as distinct but related and also correlated [45,46] concepts, researchers are now turning their attention to the relationships between eHealth literacy, health-related behaviors, and health outcomes [47,48]. Please note that, because health literacy is a key constituent of eHealth literacy and has a substantial literature, in this paper we draw on relevant research on health literacy as well as more recent studies of eHealth literacy to provide insights and inform our discussion.

In summary, most evidence (but by far not all) points in the direction that there is a negative relationship between the broader concept of health literacy and utilization of the health system, indicating that individuals with a higher level of health literacy will less frequently utilize the health care system than persons with lower levels of health literacy. Moreover, it is largely unclear what the underlying reasons of this relationship are no matter whether positive or negative. We assume these general findings and limitations also apply to eHealth literacy. The situation is equivocal enough to treat the relationship between eHealth literacy and utilization of one’s GP services and a possible network of causal relationships behind it as open questions. Consequently, the magnitude and direction of that relationship is, as research question 1 (RQ1), “How is eHealth literacy related to the number of GP consultations a person seeks?,” the starting point of our analyses.

### Mediators of the eHealth Literacy-Utilization Association

Causality between 2 variables x and y can follow 4 fundamental models: x affects y, y affects x, s affects x and y, and x affects y via m (x affects m and m affects y). The first 2 models capture direct causation in either direction. The third model is a spurious correlation that traces back the original correlation between x and y to a common cause s or any number of common causes s1, s2, s3, and so on. The fourth model divides a possible causation between x and y into 2 or more steps defined by mediating variables or mediators m1, m2, m3, and so on. All explanations summarized thus far posit mediating variables to explain the correlation. This article, therefore, is also concerned with the question of whether mediators can be found that provide a possible explanation for the correlation between eHealth literacy and number of GP visits. It does not stop at positing the role of mediators; it tests some of them.

Numerous studies report links between the general “usage” of Internet health information and various health outcomes such as improved self-care, change of decision about how to treat a condition, asking for a second opinion, improved medication compliance, and less inpatient care [49]. In relation to eHealth literacy specifically, scholars find that eHealth literacy is associated with health outcomes for the individual [50-52]. However, little research has investigated the exact nature of the relationships between eHealth literacy and particular health outcomes, especially those at the wider public health level such as utilization of the health care system.

Consequently, in this study we aim to investigate the correlation of eHealth literacy and the number of GP consultations as an aspect of utilization of the health care system. We propose and test two potential mediators: (1) Internet health information seeking and (2) gain in empowerment. Previous research explains a possible effect of health literacy on utilization of the health care system according to people’s ability to seek health information independently. For example, recent studies found strong evidence that a higher level of eHealth literacy was associated with an increase in Internet health information seeking behavior [53,54]. More importantly, people with low health literacy skills were less likely to properly evaluate health information presented on the Web [55-57]. We expect therefore that higher eHealth literacy skills are associated with an extended Web-based search for health information.

It is safe to assume that individuals with high eHealth literacy more often make use of this ability and draw more benefit from the information found. Research supports this assumption [58]; for example, Tennant et al [52] found baby boomers with high level of eHealth literacy use the Internet and social media (Web 2.0) for health-related purposes more than those with lower levels. That is to say, on commonsense grounds as well as corroborating research, we expect a positive relationship between eHealth literacy and intensity of Internet health information search. This expectation is our hypothesis (H1): Individuals who show higher levels of eHealth literacy will more often search for health information on the Internet than individuals with lower levels of eHealth literacy.

If persons with high levels of eHealth literacy do practice more and better self-education in health matters using the Internet, it remains less clear how that affects the utilization of the health care system. One could argue a negative relationship, assuming that health self-education by the Internet may spare a visit to the doctor because a good website might provide the help or advice the individual might have hoped to get from the GP. Self-education may also put a person in a position to make better judgments on the need to consult the doctor. If relatively inconsequential situations in which the individual considers a consultation are more frequent than more serious situations in which she or he does not consult the doctor, the better judgment might reduce the number of visits to one’s doctor.

In contrast, a positive relationship could also be argued, implying that successful self-educators turn to the health care system more often. One reason for this could be that their Web-based information seeking behavior makes them aware of medical conditions that are treatable and which would otherwise have just been considered a nuisance. The second reason for a positive relationship could be the explicit advice given by many medical websites to see a doctor in case of doubt whereas explicit advice not to see the doctor occurs less frequently. Hence, the better judgment through self-education might also work in direction of a positive relationship, if not consulting one’s doctor in serious conditions is the more frequent error, than seeking consultation when it is not necessary.

As both directions are possible and to some degree plausible, we formulate the relationship as research question 2 (RQ2): How does the frequency of the Internet information seeking behavior affect the utilization of the health care system?

Although there is plenty of evidence that the Internet, with its extensive availability of health information, provides many opportunities for people with high literacy skills, less is known about what the precise consequences of the additional information might be, other than being better informed. One possible effect of having Internet access to health information is that consumers are enabled to participate in decisions regarding their health [59]. Following insights from previous studies [37,60,61], we expect that consumers looking on the Web for health information will also be more empowered in the sense that they will consider themselves to be more capable of taking the proper action once they have found useful information on the health condition.

Empowerment is usually defined as the state of having or the process of acquiring mastery over one’s own life. If pertaining to health, empowerment is mastery over one’s health or the health care decisions one has to face. It can be understood as an objective state but is used in the context of health care most often in a psychological sense as the person’s subjective perception of mastery. Self-education might have objective consequences on empowerment, but as most health decisions in acute situations are made consciously, the subjective impression of such consequences becomes important. A person might, through the use of Web-based health content, become more enabled to describe their symptoms, but if she or he is also aware of that improved ability, it might be expected to be translated more easily into behavior.

As with the expectations relating to Internet information search behavior as a mediating factor, we formulate a hypothesis for the effect of eHealth literacy on a mediator, self-perceived gain in empowerment, and a research question for the relationship between the mediator and the ultimate dependent variable, utilization of the health care system. Again, the hypothesis (H2) primarily rests on plausibility: Persons who engage in more Internet health information seeking feel more empowered than persons who seek health information less often on the Internet.

Awareness of an empowering effect of information seeking has different components, among them perceived communicative abilities in dealing with GPs or other health care providers, and a form of self-assuredness in making health decisions by oneself and taking responsibility for one’s health. Better communicative abilities can be expected to result in more benefits from consultation with the GP. This suggests a positive association between self-perceived gain in empowerment and utilization of the health care system more generally. In contrast, self-assuredness in taking responsibility for one’s health rather suggests the opposite; namely, to stay away from one’s doctors. In other words, the direction of the possible association is unclear, resulting in this research question (RQ3): How does self-perceived gain in empowerment from using Internet health information affect the utilization of the health care system?

To complete the rundown of expectations, we also consider the possibility that perceived gain in empowerment is directly affected by eHealth literacy. It can be assumed that persons with a high ability to find, process, and understand information on health matters will be able to draw the benefit without necessarily reaching high values for actual information seeking. There is some support in the literature for an association between eHealth literacy and concepts related to empowerment, such as self-perceived competence in finding Web-based health information [62], aptitude, and sophistication in using such information [51]. To consider this possibility we formulate another hypothesis (H3): Persons with a high level of eHealth literacy will report higher gain in empowerment than persons with lower levels of eHealth literacy.

In summary, we investigate three paths of mediating variables that could potentially explain a relationship between eHealth literacy and utilization of the health care system: (1) via intensity of Internet health information seeking, (2) via self-perceived gain in empowerment, and (3) via both, by a path leading to intensity of Internet health information seeking and then to self-perceived gain in empowerment.

### Baby Boomers as Sampling Frame

Hypotheses and research questions were tested on baby boomers; that is, the age cohort born between the end of the Second World War and the advent of pharmaceutical contraceptives in the mid-1960s. This generation is particularly well suited for studying eHealth communication as they were relatively young at the time personal computers began to make their mark on our daily lives and they are now approaching the years when age-related troubles set in, making health a salient subject. Policy makers and health care service providers are particularly concerned at the costs and adequate provision of health care to baby boomers [63,64]. With advances in behavioral health and medicine, as the lifespan of baby boomers increases, so too will their lifetime health care costs and the pressure they exert as a cohort of significant size on the health care system. Moreover, there are mounting concerns about meeting the costs of health conditions such as diabetes and heart disease expected with the escalating number of overweight and obese baby boomers [64].

Baby boomers are increasingly using the Internet to search for and share health information [52]. For example, more than 88% of US baby boomers use a variety of digital devices to search for relevant Web-based health-related information and services, especially for increasing their knowledge of the prognosis, symptoms, and treatment options for personal health issues [65]. These emerging patterns of Web-based information seeking and sharing behaviors have immediate implications for health-related concepts such as health literacy and eHealth literacy, patient empowerment, patient autonomy, self-management, patient responsibility, and health outcomes [37,39]. Therefore, there is an urgent need to investigate baby boomers’ Web-based health information behaviors to provide a sound empirical base for designing more effective eHealth communication with them as heavy users of the health care system.

## Methods

### Sample

Data for a cross-sectional study were collected over a 4-month period in New Zealand, the United Kingdom, and the United States. The questionnaire was administered on the Web-based survey platform Qualtrics. The questionnaire was designed purposefully so there would be no “missing data;” a “not applicable” response was provided for suitable questions, and incomplete questionnaires could not be submitted. Baby boomers in each country were selected using the inclusion criteria that they were born between 1946 and 1964, and that they had used the Internet to search for and share health information in the previous 6 months. The link to the questionnaire was distributed to a representative sample stratified in terms of gender, ethnicity, education, income, and location. Approval for the research was obtained by the relevant university ethics committees before the questionnaire was pretested (6 respondents) and pilot-tested (64 respondents). The operative sample included a total of 996 persons (New Zealand, n=276; United Kingdom, n=407; United States, n=313).

Average age was 59.29 (SD 5.43). A little over half of the participants (50.1%, 499/996) were females. The modal educational level was secondary school (41.5%, 413/996); 1.2% (12/996) of the participants had a lower education, whereas 32.3% (322/996) had attended university. As to ethnicity, by far most British respondents were white (95.6%, 389/407), which was also the case for 84.8% (234/276) of the New Zealand and 79.6% (249/313) of the US sample. Almost half of the respondents (44.9%, 447/996) were employed fulltime or part-time, whereas almost a third (31.2%, 311/996) were already retired. The median income was slightly below GDP 20,000 in Britain, slightly above USD 40,000 in the United States, and between NZD 35,000-40,000 in New Zealand.

### Measures

As the measure of eHealth literacy, we used the eHealth Literacy Scale (eHEALS) as devised by Norman and Skinner [66]. It consists of 8 self-reported items that formulate self-perceived ability and confidence in gathering health information from the Internet. Items are scored on 5-point Likert scales with high scores indicating high agreement with the items and thus high eHealth literacy. The application of the measure produced reliable data (Cronbach alpha=.92, mean=3.69, SD=0.640, N=996).

Internet health information seeking behavior was measured by 4 items formulating different activities that are examples of Web-based information seeking. The items were: “I’ve looked online to try to diagnose a health condition,” “I’ve researched a health-related product or service online,” “I’ve read or watched someone else’s commentary or experience online about health-related issues,” and “I’ve read online reviews or rankings of health care services or treatments.” The corresponding question asked about the frequency of these behaviors. Items were measured on 5-point scales ranging from “never” to “very often.” The 4-item scales were averaged to achieve our measure of Internet health information seeking behavior. The scale was found to be reliable (Cronbach alpha=.80, mean=2.49, SD=0.874, N=996).

Self-perceived gain in empowerment was measured by 7 self-designed items formulating self-perceived changes attributed to the use of the Internet. The items were: “I am more aware of my health,” “I feel more in control of my health,” “I have a better understanding of the condition or disease I have,” “I feel more connected to others with a similar problem,” “I can communicate more effectively with my health professional(s),” “The quality of the relationship with my health professional(s) has improved,” and “I can make better choices about the treatment of health issues.” Items were scored on 5-point Likert scales with high scores indicating high agreement with the items and thus high self-perceived gain in empowerment. Using the measure produced reliable data (Cronbach alpha=.88, mean=3.59, SD=0.647, N=996).

Utilization of the health care system, our dependent variable was measured (as mentioned) by a single item inquiring about the number of medical consultations with one’s GP in the past year coded as 0, 1, 2, 3, 4, 5 to 9, and 10 or more. Presence of chronic disease, recoded as a binary variable from a question inquiring about 10 different chronic diseases, was used as a control variable in the ensuing analyses.

### Data Analysis

First we conducted a set of preliminary analyses including descriptive data examination, outliers, and nonnormality checks. Given this was a Web-based survey, no variables showed missing data. Second, descriptive statistics and Pearson product-moment correlation analyses were computed to determine univariate and bivariate relations among the variables in our study.

A serial mediation analysis, sometimes described as multiple-step multiple mediation [67], was conducted using the SPSS macro PROCESS [68] (model 6) with the 2 variables, Internet health information seeking behavior and self-perceived gain in empowerment, as mediators in the analysis. We used bootstrapping in the analysis to obtain bias-corrected 95% CIs for the total direct and indirect effect (ie, total mediated effect) and the specific indirect effects.

Mediation analysis used ordinary least squares path analysis. Three paths were included by which eHealth literacy may indirectly influence people to visit a GP. The first leads from eHealth literacy to GP visits via Internet information seeking behavior: those people who show higher levels of eHealth literacy are assumed to look more often for health information on the Internet and the Web-based search behavior is associated with the number of GP visits. Second, people with higher eHealth literacy levels also felt more empowered to make good health-related decisions, which in turn is assumed to affect the number of visits of the GP. Third, more eHealth literate people look for more health information on the Web, which in turn led them report a higher level of empowerment, which also is assumed to affect the number of GP visits.

## Results

Significant bivariate relationships between the main study variables—eHealth literacy, Internet health information seeking behavior, self-perceived gain in empowerment, and number of GP consultations— were detected. The strongest relationships were found between Internet health information seeking behavior and perceived gain in empowerment (*r*=.55), eHealth literacy and perceived gain in empowerment (*r*=.49), and eHealth literacy and Internet health information seeking behavior (*r*=.40), whereas the weakest association was between eHealth literacy and utilization of the health care system (*r*=.09).

**Figure 1:**
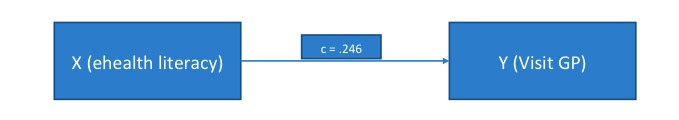
Simple regression model of GP visits on eHealth literacy.

**Figure 2:**
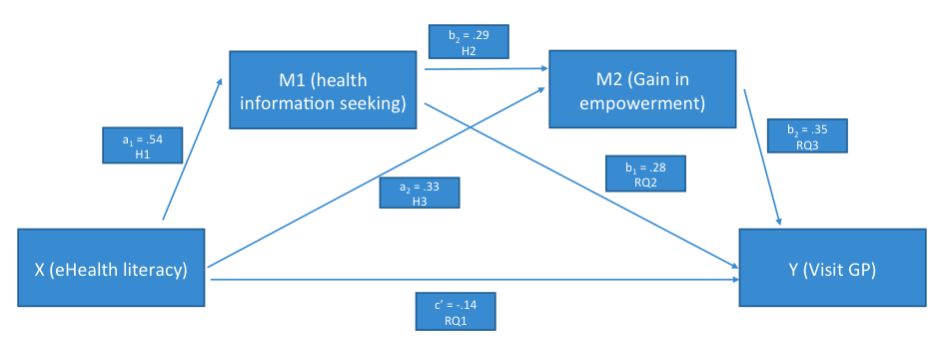
eHealth literacy serial multiple mediator model.

The paths for the full model are represented in Figures 1 and 2, the corresponding coefficients in Table 1. In the first model, number of GP consultations was predicted by eHealth literacy and the covariate chronic disease (illustrated in Figure 1), whereas the 2 mediator variables, Internet health information seeking (M_1_), and self-perceived gain in empowerment (M_2_), are excluded. The total association, c, between eHealth literacy and health system utilization is 0.009 (beta=.246, *t*_994_=2.926, *P*=.004). So, levels of eHealth literacy are very weakly but statistically significantly associated positively with health system utilization. In the second step, the 2 mediators were included in the model (illustrated in Figure 2). The first indirect path of eHealth literacy through Internet health information seeking to number of GP consultations was significant and positive (*a*_1_*b*_1_=0.1806; 95% CI=0.0993-0.2658). The second indirect path connects eHealth literacy to GP visits through the second mediator perceived empowerment; it was also significant and positive (*a*_2_*b*_2_=0.1595; 95% CI=0.0898-0.2398). Finally, the third specific path runs from eHealth literacy to Internet health information seeking to perceived gain in empowerment and to GP visits and is 0.0844 (95% CI=0.0481-0.1289); that is, significant and positive also. Taking together all specific indirect effects, that are paths *a*_1_*b*_1_, *a*_2_*b*_2_, and *a*_1_, *d*_21_, *b*_2_, the sum of all three specific indirect effects modeled amounted to 0.4245, which was different from zero as determined by the bootstrap CI which does not contain a zero (95% CI=0.3297-0.5325; Table 2). When adding the mediators to our model, the direct path from eHealth literacy to health system utilization, c, acquired a negative sign but became statistically indistinguishable from zero (*P*=.12)

With regard to our hypotheses and research questions, H1, H2, and H3 were all supported, meaning that higher eHealth literacy went along with more Internet health information seeking and self-perceived gain in empowerment, and that Internet health information seeking was associated with empowerment. RQ2 and RQ3 are answered in the positive: both Internet health information seeking and enhanced empowerment were associated with increased utilization of the health care system. RQ1 will be discussed in the following section.

**Table 1:** Regression coefficients, standard errors, and model summary information for the baby boomer serial multiple mediator model.

Antecedent	Consequence										
	M_1_(search behavior)				M_2_(gain in empowerment)				Y (general practitioner visits)		
	Coefficient	Standard error	*P* value	Coefficient		Standard error	*P* value	Coefficient		Standard error	*P* value
X (eHealth-literacy)	.558	0.041	<.001	.324		0.027	<.001	−0.139		0.091	.12
M_1_(search behavior)	–	–	–	.307		0.020	<.001	.324		0.067	<.001
M_2_(gain in empowerment)	–	–	–	–		–	–	.492		0.098	<.001
Covariate health	.036	0.025	.16	−0.004		0.016	.81	.480		0.048	<.001
Constant	.435	0.173	.01	1.606		0.107	<.001	−0.223		0.365	.54
	*R*^2^=0.1591 *F*_2, 993_=93.973 *P≤*.001				*R*^2^=0.391 *F*_3, 992_=212.159 *P≤*.001				*R*^2^=0.171 *F*_4, 991_=51.009 *P≤*.001		

**Table 2:** Total, direct and indirect effects of eHealth literacy on general practitioner visits.

Type of effect	Effect size	95% CI
Total effect of eHealth literacy on GP^a^visits	0.2859	0.1281-0.4437
Direct effect of eHealth literacy on GP visits	−0.1386	−0.3149 to 0.0377
Total indirect effects of eHealth literacy on GP visits	0.4245	0.3297-0.5325
Path 1: eHealth literacy≥search behavior≥GP visits	0.1806	0.0993-0.2658
Path 2: eHealth literacy≥search behavior≥gain in empowerment≥GP visits	0.0844	0.0481-0.1289
Path 3: eHealth literacy≥gain in empowerment≥GP visits	0.1595	0.0898-0.2398

^a^GP: general practitioner.

## Discussion

### Principal Findings

Mediation analysis usually aims at explaining some covariance between 2 variables by the influence of third variables that function as mediators. One usually finds a part of the original covariance being explained by the mediator or mediators. Our analysis is unusual in that not only was a part of the positive relationship between eHealth literacy and utilization of GP health care services explained by an indirect path, but that the indirect paths explain much more variance and eliminated the direct relationship between health literacy and utilization.

To be more precise: we investigated the way how eHealth literacy might be related with utilization of the health care system. We argued that people with higher eHealth literacy skills will turn to the Internet more often for important health information relative to those who score lower on the eHealth literacy measure, which would in turn increase their gain in empowerment, which in turn will translate into a higher number of visits of the GP. That is to say, health literacy is modeled to exert an effect on utilization of the health care system indirectly through 2 mediators: Internet health information seeking, and gain in empowerment.

The first indirect effect (path 1) is the one of eHealth literacy on Internet health information seeking. Those who show higher eHealth literacy skills were also more likely to look for health information on the Internet, and the increased search was associated with more GP visits independent of gain in empowerment. Second, another indirect effect (path 2) describes how higher level of eHealth literacy leads to a higher number of GP visits through increased search for health information on the Internet, which in turn is associated with a gain in empowerment. A third indirect effect is presented by path 3, that is, the impact of higher levels of eHealth literacy on GP visits via increased empowerment. This effect is independent of Internet health information seeking. Relative to those patients who show low levels of eHealth literacy, skillful people are more likely to consider themselves also as capable to judge whether they need to have an interaction with a GP or not, which in turn is associated with more visits.

In addition to this bundle of indirect effects, we did not find a direct effect of eHealth literacy on health care system utilization. This suggests that those who are less eHealth literate but do search for health information on the Internet and consider themselves as much empowered as those with higher levels of eHealth literacy will visit their GPs as often as those who are more literate. This effect, though, is not statistically significant if one controls also for the health status (chronic disease) of people. In other words, keeping constant Internet health information seeking behavior and empowerment, the number of GP consultations is independent of eHealth literacy.

The complete model up go posits that persons with high eHealth literacy tend to see their GP more often in as much as they seek health information on the Internet on their own or feel empowered by their Web-based information search (either directly or as a consequence of increased search behavior). This finding can be interpreted as an expression of a more dynamic element in eHealth literacy involved in the use of information and communication technologies to find relevant health information on the Web when that is necessary. It might lead to more GP visits by persons with high eHealth literacy because they have enabled themselves to make that decision.

Establishing link between eHealth literacy and utilization of health care services is the unique contribution of this research. In research into health literacy that featured an analysis structurally very similar to ours (though using performance-based measures for health literacy), Cho et al [32] found that, contrary to their expectation, neither disease knowledge, nor health behaviors, nor prevention behaviors, nor health status mediated the negative relationship between broad health literacy and utilization of the health care system. In addition to the fundamentally different results, Cho and colleagues used a different measure for utilization: hospitalization and emergency help seeking. Thus, their research does not help us to assess whether our mediators play a similar role in other studies. However, it does suggest that finding mediators of the strength we detected is unusual.

The indirect positive paths can be interpreted as indicating an improved capability in people with high eHealth literacy to distinguish serious illnesses from less consequential conditions.

In a somewhat serious health situation, people with high eHealth literacy will understand they need to act; for example, they will try to educate themselves on the Internet and consult their doctor. A heightened sense of empowerment, which is an integral part of the system of paths our results show, fits well with this kind of behavior. It might even be that, in contrast to the model presented here, a functional rather than a causal explanation of the correlation between Internet health information seeking and visits to the GP might be at work: those people with high eHealth literacy in a more serious condition already know they have to see their doctor and they use the Internet’s potential for self-education as preparation, in order to optimize the consultation.

Our results also highlight the relationship between eHealth literacy and empowerment. Schulz and Nakamoto [37] have conceptualized a model of this relationship for general health literacy and empowerment that holds that the two are not necessarily linked, as is often assumed explicitly or implicitly. Applied to eHealth literacy, the empowerment model clearly addresses the concern that high levels of empowerment in combination with lower levels of eHealth literacy mean patients are likely to make decisions that are harmful to themselves. Empowerment gives them the will to make their own decisions and low eHealth literacy prevents them from choosing the right alternative. The relationship between empowerment and eHealth literacy in this study eases that concern to some degree, as does the positive association of sense of empowerment and GP visits. Both findings emphasize a much more encouraging combination in the empowerment model: high empowerment and high eHealth literacy.

### Limitations

It might be considered a weakness of this study that it appears to treat eHealth literacy as a stand-in for the broader concept of health literacy in general. In addition to the arguments already mentioned in the text before, there is one more justification for referring to both health literacy and eHealth literacy. The measurement of health literacy is increasingly dominated by self-report measures that reach beyond the simple concept of functional health literacy but still correlate with the respective established measures covering that simple concept, such as the Short Test of Functional Health Literacy in Adults (S-TOFHLA). The measure of eHealth literacy is also based on self-reports, which establishes yet another link to the broader concept of health literacy.

Another limitation is that the utilization of the health care system is measured only with one item, number of GP consultations. A broader operationalization is to be achieved in the future.

### Suggestions for Further Research

First, further research should try and identify more variables that might mediate the relationship between health literacy and health care utilization. Second, the interpretations forwarded in the discussion (as related to the dynamic nature of health literacy and the severity of the medical condition) should be put to empirical test.

## Abbreviations

GP: general practitioner
ICT: information and communication technologies
